# Coronary Artery Disease in Women: A Comprehensive Appraisal

**DOI:** 10.3390/jcm10204664

**Published:** 2021-10-12

**Authors:** Nili Schamroth Pravda, Orith Karny-Rahkovich, Arthur Shiyovich, Miri Schamroth Pravda, Naomi Rapeport, Hana Vaknin-Assa, Alon Eisen, Ran Kornowski, Avital Porter

**Affiliations:** 1 Department of Cardiology, Rabin Medical Center, Petach Tikva 49100, Israel; orithkr@gmail.com (O.K.-R.); arthur.shiyovich@gmail.com (A.S.); hana100niki@gmail.com (H.V.-A.); Alonei1@clalit.org.il (A.E.); rkornowski@clalit.org.il (R.K.); portert@013.net (A.P.); 2 Sackler Faculty of Medicine, Tel Aviv University, Tel Aviv 39040, Israel; 3 Internal Medicine A, Meir Medical Center, Kfar Saba 44241, Israel; miripravda@gmail.com; 4 Milpark Hospital, Johannesburg 2193, South Africa; cscham@global.co.za

**Keywords:** women, sex, coronary artery disease

## Abstract

Coronary artery disease (CAD) is a significant cause of illness and death amongst women. The pathophysiology, manifestations, and outcomes of CVD and CAD differ between sexes. These sex differences remain under-recognized. The aim of this review is to highlight and raise awareness of the burden and unique aspects of CAD in women. It details the unique pathophysiology of CAD in women, cardiovascular risk factors in women (both traditional and sex-specific), the clinical presentation of CAD in women, and the range of disease in obstructive and non-obstructive CAD in women.

## 1. Introduction

Cardiovascular disease (CVD), in particular coronary artery disease (CAD), is a leading cause of morbidity and mortality amongst women [1]. Physiological and pathological cardiovascular changes are influenced by atherogenic risk factors but also by hormonal changes, unique to the course of a woman’s life. Sex (biological) and gender (socio-cultural) differences influence the clinical pattern and contribute to contemporary gender differences in the diagnosis, management, and outcomes of CVD [2].

The first women-specific clinical recommendations for the prevention of cardiovascular disease (CVD) were published in 1999, even though at that time there was little gender-specific research data [3]. Updated contemporary recommendations on CVD prevention in women emphasize the importance and impact of unique risk factors, different clinical manifestations of CVD amongst women, and treatment gaps in women’s health [1]. Despite updated guidelines and the burden of CVD on women’s health, there continues to be a deficiency of awareness about CAD in women and of the unique sex-related differences in CAD [4]. This invariably leads to a lag in the diagnosis and appropriate management of CAD in women, particularly during emergent coronary scenarios [5,6,7]. Indeed, the *Lancet* women and cardiovascular disease Commission, which aims to reduce the global burden by 2030, highlighted that CVD in women remains “understudied, under-recognised, under-diagnosed, and under-treated” [8].

The aim of this review is to highlight and raise awareness of the burden and unique aspects of coronary artery disease in women. The first section will elaborate on the unique pathophysiology of CAD in women, the second section will detail cardiovascular risk factors in women, and the third section will discuss the clinical presentation of CAD in women and the range of disease in obstructive and non-obstructive CAD in women.

## 2. Pathophysiology of CAD in Women

**Figure 1:**
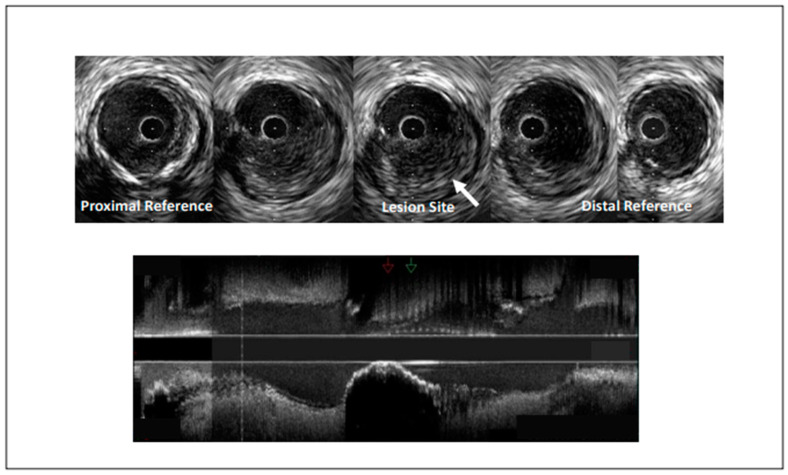
Intravascular ultrasound demonstrating coronary artery stenosis most likely due to plaque erosion.

**Figure 2:**
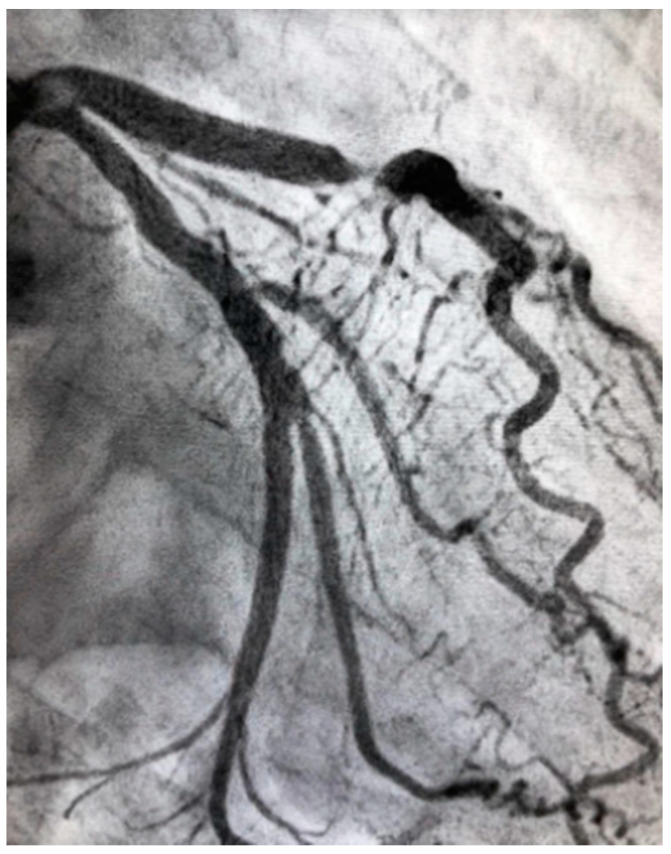
Severe atherosclerotic stenosis of the LAD in a female patient with ACS.

There is a growing body of evidence to show that vasculopathy in women with CAD is somewhat different and more severe compared to that in men. Atherosclerosis is often less extensive, and acute events are more frequently due to plaque erosion rather than plaque rupture (Figure 1 and Figure 2) [7,9]. Structurally, the coronary arteries themselves are of a smaller caliber size in women compared to men [10]. However, women and men have similar reference and lesion plaque burden, eccentricity, and calcium deposition in their atherosclerotic plaques [10].

A recent study on women undergoing coronary computer tomography angiography (CTA) provided added insight into coronary plaque assessment amongst women presenting with chest pain. Women had significantly fewer atherosclerotic plaques of all subtypes compared to men (calcified, noncalcified, and low-attenuation plaque burdens (*p* < 0.001 for all)). However, as with men, a low-attenuation plaque burden predicted future myocardial infarction events [11].

The functional aspects of the coronary vasculature are also different in women. Vascular reactivity of the endothelium and smooth muscle are responsive to sex hormones [12]. As women undergo intense hormonal influences during their lifetime, the coronary vasculature has heightened exposure to female hormones, which is believed to cause functional vascular changes. Vascular imaging studies have shown adverse changes during the menopausal transition that extend beyond the effects of aging [13]. The Women’s Ischemia Syndrome Evaluation (WISE) assessed coronary reactivity in 163 women who were referred for coronary angiography for investigation of suspected myocardial ischemia. The results showed impaired coronary vasomotor response to acetylcholine, indicating coronary endothelial dysfunction, which independently predicted adverse cardiovascular outcomes, and this was regardless of the severity of CAD [14]. There is increasing evidence to show that microvascular dysfunction is a leading adjunctive mechanism in the pathophysiology of CAD [14].

### 2.1. CAD Risk Factors

Women have both traditional and women-specific CAD risk factors. While traditional risk factors are widely recognized, specific CAD risk factors are often overlooked as risk-modifying factors.

### 2.2. Traditional CAD Risk Factors

Traditional CAD risk factors account for the majority of risk for myocardial infarction both in men and women. There is, however, evidence to suggest that some of these risk factors have a more potent effect amongst women.

1. Smoking is more detrimental to women than men, increasing the risk of myocardial infarction in women 6-fold (as opposed to men where the risk is mitigated 3-fold) [15]. There is also a compounded CAD risk between smoking and the use of hormonal treatment. This is thought to be due to prothrombotic effects. Rosenberg et al. found that heavy smoking (more than 25 cigarettes a day) increased a women’s risk of myocardial infarction by 12-fold, and this risk was compounded 32-fold in women using oral contraception [16]. Therefore, combined estrogen-progestin oral contraceptive pills should be avoided in women with a history of cardiovascular disease [17]. This compounded risk is of such concern that the use of combined oral contraceptive pills is contraindicated in women over the age of 35 who smoke and in women with severe dyslipidemia or obesity [18].
2. The prevalence and incidence of hypertension are higher in women over 60 years old. Women are less likely to receive medical treatment for hypertension and have poorer blood pressure control [5]. Furthermore, the incidence of hypertension is increased 2- to 3-fold in those taking oral contraception [19].
3. Diabetes mellitus has a more potent risk for CAD in women compared to men [5]. Diabetes mellitus also confers a higher adjusted hazard ratio (HR) of fatal CAD in diabetic women (HR = 14.74; 95% CI, 6.16–35.27) compared with diabetic men (HR = 3.77; 95% CI, 2.52–5.65). There is also a sex disparity in the intensity of cardiovascular risk reduction in women with diabetes—including poorer glycated hemoglobin levels and lower use of lipid-lowering pharmacotherapy [20].
4. Dyslipidemia is common in women. Adverse changes in the lipid profile are associated with menopausal transition [21]. SWAN (Study of Women’s Health Across the Nation) found that during menopause, women have substantial increases in total cholesterol, low-density lipoprotein, and apolipoprotein B [22] Furthermore, increases in high-density lipoprotein (HDL), which is associated with athero-protective properties in the premenopausal phase, have been found to be paradoxically associated with an increase in atherosclerosis progression in the postmenopausal phase [22]. This has been postulated to be due to changes in HDL function due to the hormonal alterative in this phase of life [23].
5. Age is a powerful predictor of CAD. While the prevalence of CAD increases with age in both men and women, the clinical presentation of CVD in women lags, on average, ~10 years behind their male counterparts [20]. Post-menopausal women more frequently have many traditional vascular disease risk conditions and these conditions cluster more frequently in them than men. These findings support the hypothesis that differences in endogenous sex hormones contribute to sex differences in CAD.

Other potentially modifiable risk factors including obesity and sedentary behaviors. These emerging risk factors are more prominent amongst women and portend a higher risk of CAD and adverse cardiovascular outcomes [5].

## 3. Women-Specific CAD Risk Factors

Women have a unique biology and unmatched phases in life characterized by hormonal changes. These include puberty, pregnancy, peripartum, and menopause. Thus, cardiovascular risk stratification in women is incomplete without thorough obstetrical and gynecological history-taking and a thorough understanding of women-specific health problems as detailed in Table 1.

### 3.1. CAD Risk Factors Associated with Pregnancy

Pregnancy creates natural stress on the cardiovascular system and is accompanied by structural and hemodynamic changes. Adverse pregnancy outcomes (APOs) are common, affecting 10–20% of pregnancies, and are related to a common etiology of placental dysfunction and maternal vascular abnormalities [29]. The disorders include the hypertensive disorders of pregnancy, pre-term birth, and intra-uterine growth restriction.

There is increasing evidence that these abnormalities can cause long-lasting detrimental changes in the cardiovascular system, which can increase the risk of CAD later in life. The pathophysiology causing this increased risk is multifactorial. The WISE-CVD study found that a history of APOs was associated with lower coronary flow, indicative of coronary microvascular dysfunction [30]. The 2021 American Heart Association scientific statement on this topic asserts that there is a strong and substantial body of evidence showing that APOs are associated with clinical cardiovascular events later in life [29]. A history of APOs is a crucial part of cardiovascular assessment in women and provides an early “window of opportunity” to assess the risk of future cardiovascular disease and/or adverse events. It is still unknown whether APOs exacerbate an underlying predisposition for the development of cardiovascular disease, or whether they initiate a cascade of proceedings that causes future cardiovascular events.

### 3.2. Hypertensive Disorders of Pregnancy

Pre-eclampsia is associated with a 2-fold increased risk of major adverse cardiovascular outcomes [18]. Zoet et al. assessed 164 asymptomatic women aged 45–55 years with previous preeclampsia and found that these women had an increased prevalence of coronary artery calcium score (CACS) (30% versus 18% in reference group; relative risk, 1.7; 95% confidence interval, 1.2–2.3) using coronary CTA imaging. These findings support the notion that these women have an increased risk of future subclinical and/or clinical coronary artery atherosclerotic disease [31]. A recent study by Wang et al. examined the association between hypertensive disorders of pregnancy and premature mortality in 88,395 parous women during 28 years of follow-up [24]. Their findings showed that either gestational hypertension or pre-eclampsia was associated with a significantly increased risk of premature death during follow-up, and this was strongest for cardiovascular-related mortality (HR: 2.26; 95% CI: 1.67 to 3.07). While those who developed subsequent chronic hypertension had a greater risk, the increased risk of mortality was seen even in the absence of subsequent chronic hypertension [24].

### 3.3. Gestational Diabetes

Gestational diabetes (GD) is defined as the development of glucose intolerance during pregnancy. It occurs in about 7% of pregnancies and is associated with a 2-fold increased risk of future cardiovascular events [18]. Women with a history of GD have also been found to have a 2-fold increased risk of CAD later in life [32]. GD is associated with an increased risk of developing subsequent and earlier-onset overt diabetes mellitus [18]. Recent data have suggested that the cardiovascular biomarker Galectin-3 increased in the first trimester amongst women who subsequently develop GD [33]. This marker may be involved in the development of cardiovascular disease in these women.

Other pregnancy complications with an increased risk of subsequent cardiovascular disease include preterm delivery, low birth weight, placental abruption, and stillbirth [18,29,34]. These findings highlight the need for long-term follow-up beyond the postpartum period in these women. These factors should be considered risk-enhancing factors [25]. On the other hand, lactation and breastfeeding have been suggested to lower a woman’s future cardiometabolic risk but future research is needed to substantiate this phenomenon [29].

### 3.4. Gynecological Conditions Unrelated to Pregnancy Polycystic Ovary Syndrome

Polycystic ovary syndrome (PCOS) is a common endocrine disorder affecting up to 10–15% of women of reproductive-age and is characterized by menstrual irregularities, polycystic ovaries, and hyperandrogenism [26]. It is a medical disorder associated with an increased cardiovascular risk. A recent Danish registry cohort study found that women with PCOS were at significantly greater CVD risk than those without PCOS (age-adjusted HR 1.20, 95% CI 1.08–1.34) [35].

### 3.5. Menopause

Women are generally at lower risk of CAD than age-matched men during their reproductive years, but this advantage disappears after menopause [5]. The risk of CVD is higher in the postmenopausal period. With increasing age, the beneficial effects of estrogen on the vasculature wanes [12,36]. Lower levels of estrogen and progesterone after menopause are believed to partially explain the increased incidence of CAD in women after menopause. The menopause transition is associated with the development of central adiposity, insulin resistance, and a pro-atherogenic lipid profile [18,27,37].

The menopausal type (natural versus surgical) and menopausal timing are associated with different cardiovascular risks. Surgical menopause (hysterectomy with or without bilateral oophorectomy) is associated with a higher CVD risk when compared with natural menopause [28]. Earlier age at natural menopause (less than 45 years) is also associated with an exceptionally increased cardiovascular risk [38].

Menopausal symptoms are not benign. Vasomotor symptoms, the hallmark of the menopausal transition, are associated with endothelial dysfunction, and thus CAD [39].

### 3.6. Menopausal Hormone Therapy (MHT)

The Women’s Health Initiative (WHI) was a primary prevention study started in 1992 involving over 160,000 post-menopausal women initiated to investigate MHT and CVD in women. MHT was initially marketed as having cardio-protective properties and as a treatment for the prevention of cardiovascular disease. However, when this was investigated, it was found that MHT of combined estrogen–progestin did not confer cardiac protection. The WHI study was stopped prematurely due to the concern that MHT may increase the risk of CAD among healthy postmenopausal women (hazard ratio for coronary heart disease of 1.24 (95% confidence interval, 1.00 to 1.54) [40]. Subsequently, there has been conflicting data. A European consensus document on this topic stated that MHT could have a potential cardiovascular benefit in women younger than 60 years old and when started within 10 years of menopause but can increase the cardiovascular risk in women with higher cardiovascular risk and after a prior cardiovascular event [18]. At present, MHT should not be used for primary or secondary prevention of cardiovascular disease per se [1].

### 3.7. Breast Cancer

Advances in the treatment of breast cancer have led to improved survival of patients. However, the morbidity and mortality of CVD are increased in these patients [5,41]. This is due to both the cardio-toxic effects of the chemotherapy and radiation-induced cardiotoxicity as well as due to the accelerated development of CAD, especially in the presence of traditional cardiovascular risk factors [42,43]. In fact, increasing survival has led to the emergence of cardiovascular disease as a major cause of morbidity and mortality in breast cancer survivors [43]. Identifying these women-specific CAD risk factors could lead to earlier recognition and increased screening of at-risk women and may have an important effect on improving outcomes [44].

## 4. Clinical Presentation of CAD in Women

Sex differences in the clinical presentation of CAD are widely reported. Women are more likely to develop angina pectoris as their first CAD symptom (47% versus 32%) and are less likely to present with an acute MI (6% versus 10%) compared to men [5].

The VIRGO study (Variation in Recovery: Role of Gender on Outcomes of Young AMI Patients) aimed to assess sex differences in the presentation and perception of symptoms among young patients (<55 years old) with MI. The results showed that the majority of women, as with men, will present with a predominant complaint of chest pain (87.0% versus 89.5%, *p* = 0.185) but women were more likely to report ≥3 associated symptoms (61.9% versus 54.8%, *p* < 0.001) [45]. Women often presented with “angina-equivalent” symptoms such as shortness of breath, palpitations, and fatigue and described a range of chest pain symptoms [2,46]. In the National Registry of Myocardial Infarction, investigations found that the proportion of patients with myocardial infarction without chest pain was significantly higher in women than men (42.0% versus 30.7%, *p* < 0.001). This was more prominent amongst younger women (<45 years old) and was associated with an increase in hospital mortality [47]. Women, themselves, are more likely to mis-attribute their pain to a non-cardiac cause, which has been shown to increase the time delay in seeking medical help [48]. The VIRGO Study also reported that women were significantly more likely to contribute their pain to anxiety/stress (20.9% versus 11.8%, *p* < 0.001) and their healthcare providers were significantly less likely to consider their symptoms as cardiac-related (53% versus 37%, *p* < 0.001) [45].

Women present with an acute myocardial infarction at an older age compared to men, and often have a greater burden of cardiovascular risk factors compared to their male counterparts. However, in recent years, a much younger age group emerged as a higher risk population. The YOUNG-MI registry investigated this population and found that younger women (<50 years old) with MI had a significantly higher proportion of diabetes, depression, and rheumatological conditions compared to their male counterparts. These women had a significantly higher all-cause mortality than men during the mean follow-up of 11.2 years [46]. One of the potential reasons that these women had poorer outcomes was that women were under-treated compared to men: They were less likely to undergo coronary angiography, less likely to undergo coronary revascularization when angiography was performed, and less likely to be treated with guideline-directed medical therapies. These findings highlight the attentiveness that physicians should have in the diagnosis and management of these patients, especially when symptoms of CAD are ambiguous or atypical among women.

### 4.1. Obstructive Versus Non-Obstructive CAD

As with men, women can present in the context of both chronic coronary syndrome (CCS) and acute coronary syndrome (ACS). In both clinical contexts, the spectrum of CAD on angiography/computer tomography imaging can be non-obstructive or obstructive.

Women with CCS are likely to have non-obstructive CAD, and this is more prevalent in women than in their male patients [13]. However, in both sexes, the majority of patients with an ACS have obstructive CAD, which is associated with increased mortality and MACE (in-hospital death, reinfarction, cardiogenic shock, or heart failure) compared to those with ACS with nonobstructive coronary arteries (MINOCA) [49]. While the incidence of acute myocardial infarction has been reported to be decreasing in the USA over time (2000–2014), this decline has slowed amongst women compared to men [50].

### 4.2. Non-Obstructive CAD

The paradox of ischemic heart disease in women has been ascribed to women having less anatomically obstructive CAD despite higher rates of myocardial ischemia and mortality compared with men [51]. This is especially pronounced in young women. The WISE study, in women with stable symptomatic CAD, demonstrated that 57% of women with symptoms and signs of ischemia had non-obstructive CAD on angiography [52]. Non-obstructive CAD is defined as <50% diameter stenosis of all major epicardial vessels and is at least twice as prevalent in women compared with men [5]. The pathophysiology suggested has been due to a more prominent process of plaque erosion and microvascular dysfunction amongst women [53]. Lack of awareness of gender differences in pathophysiology, may adversely impact CAD diagnosis in women.

In the PROMISE trial, women with stable symptoms of angina had more normal non-invasive test results for CAD than their male counterparts (61.0% versus 49.6%, *p* < 0.001). In those who underwent coronary angiography, fewer women than men had obstructive coronary lesions (40.8% versus 60.9%, *p* < 0.001). However, the investigators found that women with abnormalities on non-invasive testing were less likely to be referred for catheterization or to receive statin treatment compared to men. In the 25-month follow-up of the cohort, women overall had better outcomes (all-cause mortality, myocardial infarction, unstable angina) than their male counterparts. There was no sex-difference in outcomes in those who underwent revascularization [13]. A post-hoc analysis from the SCOT-HEART trial, in which participants with suspected CAD were evaluated with coronary CTA, found more women had normal coronary arteries (49.6% versus 26.2%) and had less obstructive CAD (11.5% versus 29.8%) [54]. However, as previously mentioned, women have more vascular dysfunction and Ischemia with No Obstructive Coronary Artery disease (INOCA) caused by microvascular disease. Therefore, these coronary CTA findings could potentially lead to fewer subsequent evaluations and fewer diagnoses of INOCA in women. The prevalence and clinical significance of small-vessel disease in patients with chest pain and normal coronary arteries or non-obstructive CHD on coronary CTA are being assessed in the Coronary Microvascular Function and CT Coronary Angiography (CorCTCA) trial [55]. This trial will perform coronary function testing during invasive angiography on these patients and will hopefully add further insight on this topic.

There has been increasing awareness of the syndromes of Myocardial Infarction/Myocardial Ischemia with Non-Obstructive Coronary Artery Disease (MINOCA/INOCA). MINOCA/INOCA represents up to 14% of all acute coronary syndromes [56]. Nearly 6% of the patients with acute MI present as MINOCA [57]. Findings from the ACTION Registry-GWTG have shown that this entity is more common in women (10.5% versus 3.4%; *p* < 0.0001) [58]. Among patients with obstructive CAD, women are reported to have higher mortality than men (3.9% versus 2.4%; *p* < 0.0001) while no sex difference in mortality has been reported in those with MINOCA (1.1% versus 1.0%; *p* = 0.84) [58].

A recent study by Reynolds et al. used coronary optical coherence tomography (OCT) and cardiac magnetic resonance (CMR) imaging to assess mechanisms of MINOCA in 145 women [59]. Multimodality imaging identified potential mechanisms in 84.5% of women with a diagnosis of MINOCA of which the majority (75.5%) were due to ischemic reasons. Nearly two-thirds of the cohort had evidence of a vascular mechanism of the myocardial infarction presentation [59]. The clinical implications are that the mechanism of MINOCA is atherothrombosis with the possible contribution of coronary artery spasm and/or endothelial dysfunction—and thus secondary prevention of atherosclerosis is vital in these patients. Adding to this is data showing that coronary arteries without focal obstructive stenosis on angiography have a significant longitudinal pressure gradient that affects coronary blood flow [60]. The myocardial vascular bed is extensive and atherosclerotic disease may be present even if it is not seen on a macrovascular epicardial level.

Importantly, despite the adverse prognosis of MINOCA, women with nonobstructive CAD and myocardial infarction are less likely to be prescribed medications for the secondary prevention of myocardial infarction [61]. The use of single antiplatelet therapy has been advocated due to the predominance of atherosclerotic disease in these patients; however, there is little evidence to support the routine use of dual antiplatelet therapy [60]. The use of dual antiplatelet therapy increases the risk of bleeding and most likely does not decrease the ischemic risk in patients without revascularization.

**Figure 3:**
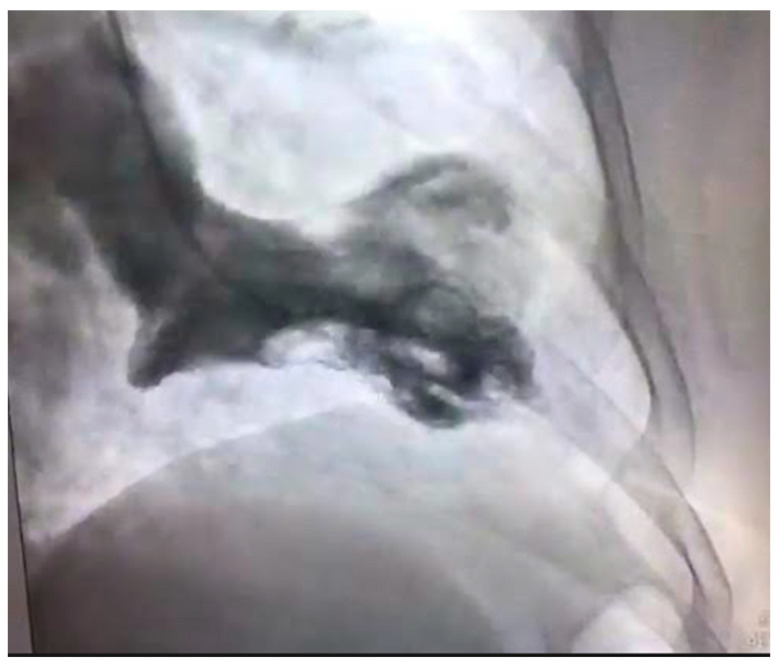
Left ventricular angiogram in a 56-year-old patient with Takotsubo Cardiomyopathy.

The differential diagnosis of MINOCA should include Takotsubo cardiomyopathy. This is a syndrome of transient ventricular apical ballooning (Figure 3). It has a female predilection with women accounting for over 90% of the cases [62]. The pathophysiology is catecholamine induced and not atherosclerotic in nature, although there is an increasing awareness of overlap syndromes of Takotsubo with CAD, with ACS playing a potentially causal role [63]. The pathophysiology of this syndrome is complex. The catecholamine surge causes a predominantly microvascular dysfunction—due to multiple factors including microvascular spasm and ensuring myocardial stunning, direct catecholamine cardio-toxicity, and an increase in the myocardial energy demand [64].

### 4.3. Obstructive CAD in Women

The majority of women presenting with ACS will have evidence of obstructive CAD [65]. Women with ACS are more likely to present with non-ST elevation myocardial infarction (NSTEMI) than with ST elevation myocardial infarction (STEMI) [45]. The etiology of ACS in women is predominantly due to atherosclerotic disease. However, entities such as spontaneous coronary artery dissection and coronary emboli should be taken into consideration. Women with STEMI have been found to have a longer time to presentation, time to diagnosis, and admission-to-treatment time compared to men [49]. The increased risk of post-MI in hospital mortality in women is predominantly attributed to those with obstructive CAD, and specifically the young age group [49,66].

### 4.4. Spontaneous Coronary Artery Dissection

**Figure 4:**
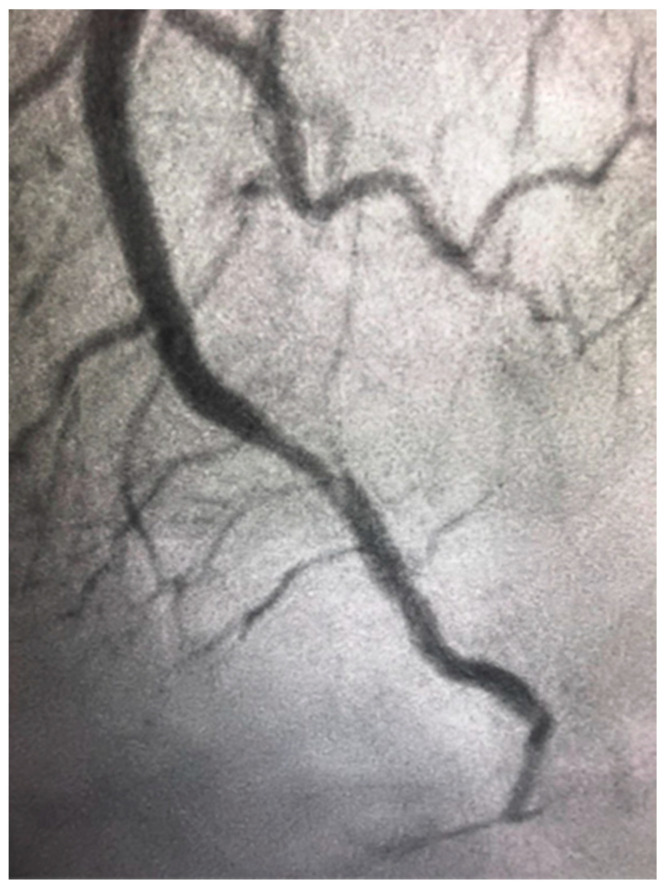
Spontaneous coronary artery dissection in the distal left anterior descending artery in a 46-year-old woman presenting with ST elevation myocardial infarction.

Spontaneous coronary artery dissection (SCAD) is due to spontaneous separation of the coronary arterial wall, creating an intramural hematoma that occludes the coronary lumen, and it is not associated with atherosclerosis (as shown in Figure 4). It overwhelmingly occurs in women. SCAD accounts for up to 4% of myocardial infarction in women and it is even more frequent among young women [67]. SCAD has a predominance in young women and is the most common cause of pregnancy-associated MI (43%) [67]. It is vital that the treating physician take SCAD into consideration when treating women with ACS. Firstly, it is associated with unique risk factors and conditions, such as a high prevalence of fibromuscular dysplasia, which need further systemic workup. Secondly, intravascular imaging should be used in some cases during coronary angiography in order to visualize the arterial wall and aid in diagnosis. Thirdly, SCAD has different therapeutic and prognostic implications compared with atherosclerotic coronary disease. It is associated with lower technical success during PCI and suboptimal outcomes, and the preferred management of SCAD is conservative in many cases [67]. Furthermore, there is an increased risk of periprocedural complications most likely because the underlying coronary artery wall is inherently weak and prone to iatrogenic dissection and propagation of SCAD with attempts to perform PCI.

## 5. Treatment Gaps

A large body of data has shown that women with ACS are less likely to be treated with guideline-directed medical therapy, less likely to undergo PCI, and more likely to have a time delay to reperfusion [2,46]. Guideline recommendations endorse the idea that the management of ACS should be the same for men and women and that PCI is the preferred reperfusion strategy [2,68]. The management of an acute myocardial infarction should be PCI in most patients. As with men, women have better outcomes with early invasive management with reduced mortality and recurrent myocardial infarction and/or ischemic events [2]. Indeed, there is evidence to show that amongst patients with ACS underdoing PCI, there are no sex differences in clinical endpoints after correcting for the advanced age and increased burden of comorbidities amongst women [69].

The sex disparities in treatment are specifically seen in women with MINOCA, who are less likely to be prescribed medications for the secondary prevention of MI [70]. This sex disparity is prominent in women under the age of 55 years who are less likely to receive appropriate medical therapy following an ACS event. Importantly, this has been shown to be mainly due to lower treatment initiation, rather than lower treatment adherence [71].

### 5.1. Bleeding Risk in Women

Women have an increased bleeding risk with antiplatelet and antithrombotic agents. This has been suggested to be due to gender differences in pharmacokinetic and pharmacodynamic aspects [72].

The TWILIGHT study assessed early aspirin withdrawal with the continuation of ticagrelor after percutaneous coronary intervention (PCI) in patients at high risk for bleeding or ischemic events. Early aspirin withdrawal with the continuation of ticagrelor was associated with a lower bleeding rate amongst both sexes with no increased risk of ischemic events [73]. Thus, this treatment strategy could potentially have a specific advantage in women.

### 5.2. Coronary Artery Bypass Surgery

Women needing surgical revascularization are generally older than their male counterparts and have more co-morbidities. Even when adjusting for cofounding factors, women have increased in-hospital mortality compared to men and suffer from more postoperative complications such as renal failure and neurological complications. These sex-differences are more pronounced in younger women undergoing coronary bypass surgery (<50 years old) [74].

### 5.3. Cardiac Rehabilitation Programs

Participating in Cardiac Rehabilitation (CR) programs has demonstrated significant reductions in reducing repeat hospitalizations and cardiovascular mortality [75,76]. According to observational studies, women achieve at least similar, if not greater, improvements than those of men participating in CR [77]. Patients following a myocardial infarction should be referred to CR as a part of international practice guideline recommendations [72]. However, women are less likely to be referred to CR and adhere to CR programs than men [75,78]. Some of the reasons suggested for this are socio-economic reasons, women having time restraints as the primary caretakers of the home, and low rates of appropriate physician referral [77,78,79]. Strategies to increase women’s referral and adherence to CR are crucial and should be implemented.

### 5.4. Prognosis

Women with CCS have fewer atherosclerotic diseases compared to men and data have shown that their prognosis is better than their male counterparts. However, compared to men, women with ACS and those after coronary revascularization have longer hospitalizations, higher in-hospital mortality, and increased readmissions during the first 30 days after hospitalization [2]. Female sex has been shown to be an independent poor prognostic factor in the clinical context of STEMI [49]. Mechanical complications and subsequent heart failure are more likely to develop in women after an acute myocardial infarction [2]. Encouragingly, there is evidence to show that over the last 20 years, the rate of 30-day major adverse cardiac events and in-hospital complications amongst women with the acute coronary syndrome is decreasing [80].

## 6. Conclusions

In summary, cardiovascular disease and CAD are among the leading causes of morbidity and mortality in women. In recent years, understanding that manifestations and outcomes of cardiovascular syndromes differ between genders has raised. Nevertheless, important issues such as women-specific risk factors, hormonal influences, and different pathophysiology mechanisms have been under-researched, under-recognized, and under diagnosed. There is an unmet need for better research, diagnosis, and treatment, for improving women’s cardiovascular outcomes and well-being at large.

## Figures and Tables

**Table 1:** Women-specific CAD risk factors.

CAD risk factors associated with Pregnancy	Adverse pregnancy outcomes (APOs)	APOs include the hypertensive disorders of pregnancy, pre-term birth and intra-uterine growth restriction. APOs are associated with microvascular dysfunction and a higher risk of cardiovascular events later in life [19]
	Hypertensive disorders of pregnancy	Women with pre-eclampsia have an increased risk of future subclinical coronary artery atherosclerosis [21].
	Gestational Diabetes (GD)	Women with a history of GD have also been found to have a 2-fold increased risk of CAD later in life [23].
Gynecological conditions unrelated to pregnancy	Polycystic ovary syndrome (PCOS)	PCOS is associated with a greater cardiovascular risk [24].
	Menopause	The risk of CVD is higher in the postmenopausal period. Surgical menopause and earlier age at natural menopause are associated with an increased cardiovascular risk [25,26].
	Menopausal Hormone Therapy (MHT)	MHT could have a potential cardiovascular benefit in women younger than 60 years old and when started within 10 years of menopause but can increase the cardiovascular risk in women with higher cardiovascular risk and after a prior cardiovascular event [16].
Breast Cancer		cardio-toxic effects of the chemotherapy and radiation-induced cardiotoxicity as well as due to accelerated development of CAD [27,28].
